# Prevalence of Avian Influenza Virus in Atypical Wild Birds Host Groups during an Outbreak of Highly Pathogenic Strain EA/AM H5N1

**DOI:** 10.1155/2024/4009552

**Published:** 2024-07-29

**Authors:** Jourdan M. Ringenberg, Kelsey Weir, Lee Humberg, Carl Voglewede, Mitch Oswald, J. Jeffrey Root, Krista Dilione, Evan Casey, Michael Milleson, Timothy Linder, Julianna Lenoch

**Affiliations:** ^1^ U.S. Department of Agriculture Animal and Plant Health Inspection Service Wildlife Services National Wildlife Disease Program, Fort Collins 80521, Colorado, USA; ^2^ U.S. Department of Agriculture Animal and Plant Health Inspection Service Wildlife Services, West Lafayette 47907, Indiana, USA; ^3^ U.S. Department of Agriculture Animal and Plant Health Inspection Service Wildlife Services, Springfield 62711, Illinois, USA; ^4^ U.S. Department of Agriculture Animal and Plant Health Inspection Service Wildlife Services National Wildlife Research Center, Fort Collins 80521, Colorado, USA; ^5^ U.S. Department of Agriculture Animal and Plant Health Inspection Service Wildlife Services, Gainesville 32601, Florida, USA

## Abstract

The global outbreak of highly pathogenic avian influenza (HPAI) H5N1 Eurasian lineage goose/Guangdong clade 2.3.4.4b virus that was detected in North America in 2021 is the largest in history and has significantly impacted wild bird populations and domestic poultry across the continent. Synanthropic birds may play an important role in transmitting the virus laterally to other wild bird species and domestic poultry. Understanding the dynamics of HPAI in atypical, or nonreservoir, wild bird hosts may help inform management decisions and potential risk factors to both wild and domestic bird populations. Following the confirmation of infections of HPAI H5N1 in domestic poultry at two commercial premises in Indiana, United States, we sampled and tested 266 Columbiformes and Passeriformes birds and found no detections of the virus at either location. We further queried laboratories within the National Animal Health Laboratory Network for avian influenza (AI) virus diagnostic test results for wild birds submitted from morbidity/mortality events, for a total of 9,368 birds tested across eight orders and 1,543 avian influenza virus detections between February 2022 and March 2023. Query results were assessed for viral prevalence by taxonomic group and suggested that the virus most often was observed in predatory and scavenging birds. The highest prevalence was observed in raptors (0.2514), with prevalence rates in exclusively scavenging *Cathartidae* reaching up to 0.5333. There is evidence that the consumption of infected tissues is a key pathway for transmission of AI viruses in predatory and scavenging birds. Although detections were found in nonpredatory synanthropic birds, including orders Columbiformes and Passeriformes, the risk of transmission from and between these groups appears comparatively low. Understanding the dynamics of AI viruses in synanthropic bird orders during the global HPAI H5N1 outbreak in wild bird populations can provide pertinent information on viral transmission, disease ecology, and risk to humans and agriculture.

## 1. Introduction

The global outbreak of highly pathogenic avian influenza (HPAI) H5N1 Eurasian lineage goose/Guangdong (Gs/GD) clade 2.3.4.4b virus (hereafter H5N1) that was detected in North America in 2021 (hereafter the outbreak) is the largest in history and has impacted wild bird populations and domestic poultry significantly across the continent. The first known infection of H5N1 in North America occurred in a wild great black-backed gull (*Larus marinus*) from Newfoundland and Labrador Province, Canada, in November 2021 [1]. In January 2022, H5N1 was reported in apparently healthy wild waterfowl from North Carolina and South Carolina, United States (U.S.), and since then, there have been over 9,500 confirmed HPAI H5 detections in over 170 wild bird species across 19 avian orders in 49 states [2]. As HPAI H5Nx subtypes continue to circulate throughout Eurasia and the Americas [3, 4], the migratory nature of wild birds introduces the risk of recombination and reassortment and the introduction of new strains into North America [5, 6, 7]. Understanding AI virus dynamics in wild bird species can help inform management decisions for wild bird populations and the commercial poultry industry.

The avian orders Anseriformes (ducks, geese, and swans) and Charadriiformes (shorebirds, gulls, and terns) act as the primary reservoir hosts of avian influenza (AI) viruses in the wild [8, 9]. Waterfowl play a significant role in the transmission of AI viruses, but they often present as asymptomatic and survive viral infection [10, 11]. While methods of viral transmission are well understood in these orders, less is known about the role alternative avian host species play in transmitting AI viruses across the landscape during an HPAI virus outbreak. Understanding AI virus dynamics in different orders may help identify introduction and transmission pathways which can be used to determine areas with greater risk of HPAI virus infection to alternative avian hosts, threatened and endangered species, and domestic poultry.

Following the initial detection of HPAI H5N1 in a commercial turkey facility in Indiana (IN), U.S., in February 2022, detections in domestic poultry (commercial and backyard flocks) have occurred alongside wild bird detections throughout the course of the outbreak with confirmed infections in 47 states [12]. Exact mechanisms of H5N1 transmission from wild birds to poultry are speculative, but bridge hosts, which are nonmaintenance host species that can transmit pathogens from reservoir species to domestic poultry through shared resources (e.g., water, crops, feed), could play a vital role [13, 14]. Synanthropes, or species that are ecologically associated with human populations and regularly utilize anthropogenically modified environments, may act as bridge hosts [15].

Known broadly for their synanthropic behavior, species in the order Columbiformes (doves and pigeons) often have been the subject of AI virus research [15] and investigated as potential bridge hosts in transmitting AI viruses between migratory birds and poultry or between poultry facilities during disease outbreaks [16]. Experimental infections of rock doves (*Columba livia*) have shown their role in AI virus transmission is likely via fomite or mechanical routes, and when they do shed virus, the quantities and time frames of shedding are limited [15, 17]. While the risk for transmission to domestic poultry is low, there is evidence that some AI virus strains can spread from Columbiformes to other avian species and cause infection [15].

The order Passeriformes contains several families of birds that demonstrate synanthropic behavior, including *Corvidae* (crows, jays, magpies, and ravens), *Fringillidae* (finches), *Hirundinidae* (swallows), *Icteridae* (blackbirds and grackles), *Passeridae* (Old World sparrows), *Sturnidae* (starlings), and *Turdidae* (robins and thrushes) [15]. Many species within these families commonly are found on farms and have the potential to act as bridge hosts. Susceptibility to AI viruses has been shown both experimentally and in the wild in several Passeriformes species. In a review evaluating AI virus infection rates in wild birds globally, researchers calculated a 0.0206 prevalence rate for all Passeriformes tested [18]; however, evidence of AI virus susceptibility differs between species.

Many *Corvidae* species are omnivorous, opportunistic foragers, and keen scavengers that commonly are attracted to carcasses accessible on farms. Studies evaluating both natural and experimental infections of HPAI viruses in *Corvidae* suggest they may play an important ecological and epidemiological role in HPAI H5 virus dynamics. In South Korea in 2003–2004, H5N1 was detected in Korean magpies (*Pica pica sericea*) found dead at a poultry facility [19], and investigations of large-billed crow (*Corvus macrorhynchos*) mortalities closely associated with an H5N1 domestic poultry outbreak in Japan in 2004 demonstrated their susceptibility to infection [20]. Experimental inoculations of corvids, including house crows (*C. splendens*) and rooks (*C. frugilegus*), have suggested that infection can result in clinical signs, seroconversion, viral shedding, and mortalities, and corvids have previously been identified as a large risk factor for virus dispersal [21, 22, 23].

Species within non-*Corvidae* families in the order Passeriformes often are colloquially referred to as songbirds, but distinct differences between them have important implications for AI virus transmission. Of species in the *Fringillidae* family, house finches (*Haemorhous mexicanus*) commonly display synanthropic behavior, yet the few assessments of their susceptibility to AI viruses have found low prevalence rates, suggesting low transmission risk [15]. While the insectivorous diet of *Hirundinidae* species could decrease their likelihood of interacting with poultry or shared resources [15], their global abundance and occupancy on farms stress the importance of understanding their role in AI virus ecology [15]. Studies have demonstrated swallows' susceptibility to AI viruses [24, 25] and potential to act as bridge hosts [26, 27]. Several *Icteridae* species are a common presence on farms [15], but results of AI virus transmission are mixed, and more research is needed to better understand the role they play in spillover to poultry. Within the *Passeridae* family, sparrows are susceptible to many AI viruses of which they can shed high levels and transmit to poultry [15]. Two studies that experimentally inoculated: (1) tree sparrows (*Spizelloides arborea*) with four HPAI H5Nx virus strains [28] and (2) house sparrows (*Passer domesticus*) with HPAI H5N1 [17] found both species to be highly susceptible. European starlings (*Sturnus vulgaris*), the most common, widespread synanthrope in the *Sturnidae* family, often flock to farms for food resources and nesting sites in groups so large that even small amounts of viral shedding by individuals collectively could cause AI virus spillover to poultry [15, 29, 30]. Starlings sampled and tested for AI viruses across 14 studies showed a 0.018 prevalence rate, but their role in transmission may be strain dependent [15]. Within the *Turdidae* family, during a surveillance study conducted in passerines across the U.S., AI viruses were detected in American robins (*Turdus migratorius*) and Swainson's thrush (*Catharus ustulatus*) at rates of 0.0376 and 0.0377, respectively [31]. Further, an experimental study inoculating American robins with HPAI H5Nx viruses found 0.8800 prevalence [32]. Ultimately, songbird susceptibility to AI viruses is variable, and more work is needed to evaluate the spillover risk to poultry.

The order Galliformes (pheasants, turkeys, grouse, and quail) often exhibits synanthropic behavior and evidence has shown that many species in this family are susceptible to and can shed AI viruses [15]. Many Galliformes that have been studied are domesticated and raised in backyard or gamebird farms, so less is understood about contact frequency and AI virus dynamics between wild and domestic individuals. A serosurvey in Italy of 219 free-living pheasants (*Phasianus colchicus*) found a 0.1230 prevalence rate but detected no antibodies to low-pathogenic avian influenza (LPAI) virus H5 subtypes [33]. A similar study of hunter harvested, wild-captured bobwhite quail (*Colinus virginianus*) in Texas, U.S., found 1.4% positive and 7.6% suspect for AI viruses [34].

Feeding behavior of avian scavengers and predators provides the opportunity for contact with HPAI virus-infected carcasses or prey, and exposures and infections have been detected in Accipitriformes (hawks and eagles), Cathartiformes (New World vultures), Falconiformes (falcons), and Strigiformes (owls) [15]. Passive surveillance and diagnostics following the HPAI H5Nx outbreak in the U.S. in 2014–2015 found raptors (hawks, eagles, and owls) to be highly susceptible to HPAI H5 clade 2.3.4.4 viruses, with an overall positivity rate of 52.4% [10, 35]. Hall et al. [36] found American kestrels (*Falco sparverius*) to be highly susceptible to H5N1 with 100% mortality rate of experimentally inoculated birds. Additionally, following the initiation of the EA H5N1 outbreak in the southeastern U.S. coast in late 2021, researchers observed high rates of reproductive failure in bald eagles throughout the area [37]. However, other studies have noted low prevalence in raptor species. Findings in an examination of raptors in Oklahoma, U.S., found only 0.0160 prevalence in red-tailed hawks (*Buteo jamaicensis*) [38]. Evidence of AI virus exposure in raptors that specifically scavenge or prey upon aquatic birds was found in bald eagles (*Haliaeetus leucocephalus*; 5.1%), with negligible evidence of exposure in peregrine falcons (*F. peregrinus*; 0.2%), great horned owls (*Bubo virginianus*; 1.2%), and Cooper's hawks (*Accipiter cooperii*; 1.0%), and zero evidence of exposure in vultures [39]. Regardless, there is strong evidence of susceptibility to highly pathogenic and other AI viruses in these orders, and understanding their prevalence throughout the outbreak can help add to the body of knowledge and provide management insight [40, 41].

The objectives of this study were (1) to assess the presence of HPAI viruses in synanthropic birds captured around H5N1-positive commercial poultry premises in response to the initial detection in domestic poultry in the U.S. and (2) to evaluate the prevalence of AI viruses in synanthropic bird orders during an HPAI outbreak. To address the first objective, we initiated a surveillance project to sample synanthropic bird species around HPAI-affected commercial poultry premises and tested for the presence of AI viruses. To address the second objective, we evaluated data from the National Animal Health Laboratory Network (NAHLN) on wild bird species submitted for AI virus diagnostic testing as part of morbidity/mortality (M/M) investigations. In this study, we report results from the targeted surveillance project, compare prevalence rates of AI viruses in several avian orders submitted from M/M investigations from February 2022 to March 2023, and provide the total number of HPAI EA H5 positive birds confirmed at the National Veterinary Services Laboratories (NVSL) from avian orders of interest.

## 2. Materials and Methods

### 2.1. Targeted Surveillance

Synanthropic bird species were sampled at two adjacent commercial domestic turkey farms with confirmed HPAI H5N1 in Dubois County, IN, U.S., in February 2022. Samples were collected in accordance with the U.S. Department of Agriculture (USDA) Wild Bird Avian Influenza Surveillance Field Procedures Manual (Summer FY2022 to Winter FY2023) and within the guidelines and regulations set forth by the U.S. Fish and Wildlife Service (USFWS) under permit number MB124992. All samples were collected with permission of the farm owners. Sampling of wild birds began approximately 2 weeks following virus detection and the initiation of poultry depopulation. A clean and dirty line was established on both premises, requiring all people, vehicles, supplies, and equipment to be fully cleaned and disinfected prior to crossing from the dirty side to the clean side. Traps were deployed to target European starlings, house sparrows, and rock doves. Five trap designs were used: custom three-hole wooden nest box traps composed of vertically stacked Sherman traps (H. B. Sherman Traps, Inc., Tallahassee, Florida, U.S.); custom-made PVC single-hole nest box traps with PVC caps and a single catch trap door (Van Ert Enterprises, Decatur, Iowa, U.S.); custom portable single-axle trailer drop-in starling decoy traps; baited walk-in traps with funnels; and decoy, walk-in pigeon traps (Tomahawk Live Trap, Hazelhurst, Wisconsin, U.S.). Traps were set within the perimeter of the infected farms and placed around poultry barns, grain bins, feed silos, other farm structures, and suspected avian movement corridors on edges of natural or agriculturally modified habitats. Traps were set every morning on each site and checked within 24 hr for a total of 18 days. Traps were baited with commercial bird seed, dry cat food, and corn. Traps were disinfected with Virkon™ S (LANXESS, Pittsburgh, Pennsylvania, U.S.) before transferring to a new location.

All captured species were identified by field biologists. Upon capture birds were immediately euthanized via cervical dislocation and subsequently sampled. Oropharyngeal and cloacal swabs (Harmony Lab and Safety Supplies, Grove Garden, California, U.S.) were collected from all captured birds. Both swabs were pooled into a single tube containing 1.5 mL of PrimeStore® Molecular Transport Medium (MTM; EKF Diagnostic, Barleben, Germany) and were shipped to the Veterinary Diagnostic Laboratory at Colorado State University within 3 days to maintain sample integrity. Nucleic acids were extracted from samples following standard extraction protocols, and a general influenza Type A rRT-PCR assay targeting the conserved region of the avian influenza matrix gene was performed [42, 43]. Per NAHLN protocol, only samples with a cycle threshold (Ct) value greater than 0 by general influenza Type A rRT-PCR assay are further tested by the H5 and H7 rRT-PCR subtyping assays [43].

### 2.2. Morbidity and Mortality Investigations

Morbidity and mortality (M/M) investigations were conducted across numerous species of birds that appeared sick, moribund, or dead due to suspected exposure to HPAI H5N1 within the conterminous U.S. and Alaska throughout the outbreak. Tracheal, cloacal, and/or oropharyngeal swab(s); whole carcasses; or tissue samples were collected opportunistically by state agencies, federal agencies, universities, and rehabilitation facilities. Sampling methodology may have differed depending on the collecting state, agency, or facility in terms of number of birds sampled at each M/M event and type(s) of samples collected. No more than five samples were collected from any individual M/M event. Samples were submitted to labs in the NAHLN for diagnostic testing, which included a general influenza Type A rRT-PCR assay for all samples and further H5/H7 subtyping assays for samples with a Ct value greater than 0 [42, 43].

We queried all laboratories in the NAHLN and provided a standardized spreadsheet to be completed with a list of species across multiple taxonomic groups. We focused on groups that most commonly exhibit synanthropic behavior but excluded known reservoir host and other aquatic bird species. Labs recorded the number of each species tested from 1 February 2022–31 March 2023, and the number of non-negative samples as determined by the general influenza Type A rRT-PCR assay [42]. We compiled responses from each lab and calculated the total number of each species tested. We grouped species by family and order, calculated the prevalence of AI virus detections in each group, and calculated 95% confidence intervals for each species and family (Table S1). Known captive and domestic birds were excluded from the dataset.

### 2.3. Confirmatory Testing of HPAI H5 and H5N1 Detections

Lastly, we report the total number of EA H5 and EA H5N1 detections in avian orders of interest from the wild bird HPAI detection dataset between 1 February 2022 and 31 March 2023 [2]. The wild bird HPAI detection dataset is maintained by the USDA, encompasses all confirmed EA H5 and H5N1 detections in wild birds in the U.S. since the outbreak began, and is reported publicly and to the World Organisation of Animal Health. Samples were submitted as part of M/M events by state agencies, federal agencies, universities, and rehabilitation facilities either to a lab in the NAHLN for initial screening or directly to the NVSL. Samples first screened at the NAHLN labs with a resulting non-negative Ct value, as determined by the general influenza Type A rRT-PCR assay, were forwarded to the NVSL for confirmatory testing. Confirmatory testing at the NVSL included an rRT-PCR assay targeting Eurasian lineage Gs/GD H5 clade 2.3.4.4b (SEPRL; Real-Time RT-PCR Assay for the Detection of Goose/Guangdong lineage Influenza A subtype H5, clade 2.3.4.4; NVSL-WI-1732), as well as an N1 subtyping rRT-PCR assay (SEPRL; Real-Time RT-PCR Assay for the Detection of Eurasian-lineage Influenza A Subtype N1; NVSL-WI-1768). Samples submitted from birds belonging to the orders Anseriformes, Charadriiformes, Pelecaniformes, Suliformes, and Gruiformes were removed from the wild bird NVSL dataset, as they were not the focus of our study.

## 3. Results

### 3.1. Targeted Surveillance

Samples were obtained from a total of 266 wild synanthropic birds across eight species from two adjacent commercial turkey farms with confirmed HPAI H5N1 in Dubois County, IN, U.S. (Table 1). Samples were obtained from the families *Columbidae* (44), *Icteridae* (81), *Passeridae* (89), and *Sturnidae* (52). None of the 266 individuals tested positive for influenza A virus by rRT-PCR from pooled cloacal and oral swabs, resulting in zero prevalence of AI virus. With no Ct values greater than 0, targeted surveillance samples were not eligible for further H5/H7 subtyping.

### 3.2. Morbidity and Mortality Investigations

**Figure 1:**
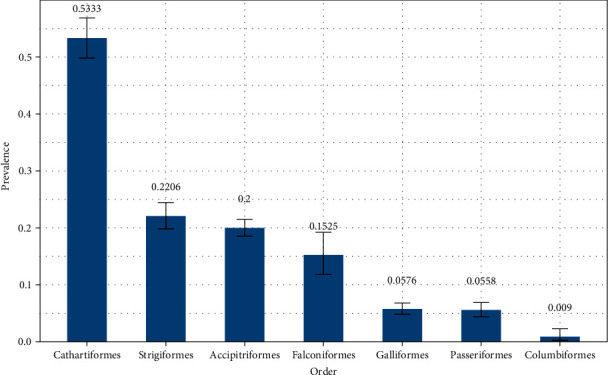
Prevalence of avian influenza A viruses in avian orders submitted to the NAHLN as part of morbidity/mortality investigations between 1 February 2022 and 31 March 2023. Exact prevalence is shown above each bar and error bars are included to display 95% confidence intervals for each order.

Out of the 48 labs queried in the NAHLN, 32 labs (67%) provided influenza A virus diagnostic testing data broken down by species. Responses from the NAHLN labs were provided as summaries of the number of individuals within a species diagnostically screened by the general influenza Type A rRT-PCR and the number of resulting non-negative samples. Due to limits in data availability, we only report general influenza Type A rRT-PCR results for samples tested at the NAHLN. Results from the NAHLN labs show a total of 9,368 birds tested and 1,543 AI virus detections observed (prevalence of 0.1778; Table S1). Prevalence rates were highest in Cathartiformes followed by Strigiformes, Accipitriformes, Falconiformes, Galliformes, Passeriformes, and Columbiformes (Figure 1).

### 3.3. Prevalence of AI Virus by Orders of Synanthropic Birds

#### 3.3.1. Pigeons, Doves: Order Columbiformes, Family Columbidae

Out of the 443 samples collected from the family *Columbidae*, four tested positive for AI viruses resulting in a prevalence of 0.0090 (Table S1). Mourning doves (*Zenaida macroura*) and rock doves accounted for 76% of *Columbidae* samples and all AI virus detections, with a higher prevalence rate in mourning doves (0.0217) than rock doves (0.0082).

#### 3.3.2. Crows, Ravens, Jays, and Magpies: Order Passeriformes, Family Corvidae

Of the 532 *Corvidae* tested, 66 were positive for AI viruses, resulting in a total prevalence of 0.1240 (Table S1). Prevalence was highest in common ravens (*Corvus corax*; 0.2358) followed by fish crows (*C. ossifragus*; 0.2083), magpies (*Pica* spp; 0.1429), and American crows (*C. brachyrhynchos*; 0.0997).

#### 3.3.3. Songbirds: Orders Passeriformes and Piciformes

A total of 889 samples were obtained from the orders Passeriformes and Piciformes, 13 of which tested positive for AI viruses, resulting in a total prevalence of 0.0150 (Table S1). Of the families tested, AI virus was detected in *Fringillidae*, *Hirundinidae*, *Icteridae*, *Passerellidae*, *Passeridae*, and *Turdidae*. Prevalence was highest in *Hirundinidae* (0.1429), with five total detections in swallow species (*Tachycineta bicolor* and *T. thalassina*). *Fringillidae* had a prevalence of 0.0426, with one detection each in an American goldfinch (*Spinus tristis*) and a pine grosbeak (*Pinicola enucleator*). *Passerellidae* had a prevalence of 0.0333, with one detection in a dark-eyed junco (*Junco hyemalis*). *Icteridae* yielded a prevalence of 0.0250, with one detection each in a boat-tailed grackle (*Quiscalus major*), a common grackle, and a red-winged blackbird. Lowest detected prevalence rates were observed in the families *Passeridae* (0.0061), with one house sparrow detection, and *Turdidae* (0.0040), with one American robin detection.

#### 3.3.4. Raptors: Orders Accipitriformes, Cathartiformes, Strigiformes, and Falconiformes

Of the 5,306 raptor samples submitted for testing, 1,334 were positive for AI viruses, resulting in a total prevalence of 0.2514 (Table S1). Raptor species accounted for 57% of all samples submitted. Prevalence was highest in the *Cathartidae* family (0.5333) followed by *Strigidae* (0.2318), *Accipitridae* (0.2044), *Falconidae* (0.1525), and *Pandionidae* (0.0488). The highest prevalence rate was observed in black vultures (*Coragyps atratus*; 0.6788). Of the remaining *Cathartidae*, prevalence rates in turkey vultures (*Cathartes aura*; 0.3925) and unspecified *Cathartidae* (0.3529) were higher than that of California condors (*Vultur gryphus*; 0.0375). In the *Accipitridae* family, prevalence was highest in rough-legged hawks (*Buteo lagopus*; 0.5000) followed by red-tailed hawks (0.2584), bald eagles (0.2557), Swainson's hawks (*B. swainsoni*; 0.2105), unspecified eagles (0.1538), unspecified hawks (0.1282), and red-shouldered hawks (*B. lineatus*; 0.1117). The prevalence rates of the remaining *Accipitridae* species tested were below 0.1000. Of the species within *Falconidae* with a sample size greater than one, the peregrine falcon exhibited the highest prevalence of 0.3108. The remaining *Falconidae* species had prevalence rates below 0.1000. Osprey (*Pandion haliaetus*), the only species within *Pandionidae*, had a prevalence rate of 0.0488. Prevalence in the *Strigidae* family was highest in great horned owls (0.3836) followed by short-eared owls (*Asio flammeus*; 0.3333) and snowy owls (*Bubo scandiacus*; 0.3214). Barred owls (*Strix varia*), eastern screech-owls (*Megascops asio*), long-eared owls (*A. otus*), and unidentified *Strigidae* species all had prevalence rates below 0.1000.

#### 3.3.5. Pheasants, Turkeys, Grouse, and Quail: Order Galliformes

Out of the 2,187 Galliformes species submitted for testing, 126 tested positive for AI viruses, resulting in a total prevalence of 0.0576 (Table S1). Prevalence rates within the *Phasianidae and Odontophoridae* families were 0.0860 and 0.0040, respectively. Of the species tested within *Odontophoridae*, prevalence was highest in greater sage grouse (*Centrocercus urophasinus;* 0.2000) followed by ring-necked pheasants (*Phasianus colchicus;* 0.1330), unidentified pheasants (0.0905), ruffed grouse (*Bonasa umbellus;* 0.0667), and wild turkeys (*Meleagris gallopavo; 0.0643*).

### 3.4. National Veterinary Services Laboratories

A total of 2,121 samples from six synanthropic orders of interest were confirmed as the Eurasian lineage Gs/GD H5 clade 2.3.4.4b subtype at the NVSL between 1 February 2022 and 31 March 2023 (Table 2). Approximately 92% of the samples (1,942) originated from raptors: 840 Accipitriformes, 671 Cathartiformes, 61 Falconiformes, and 370 Strigiformes. Both EA H5 and EA H5N1 confirmed detections are presented for congruency with what is reported to the World Organisation of Animal Health.

## 4. Discussion

### 4.1. Targeted Surveillance

Based on rRT-PCR results, we did not detect any AI viruses in the 266 wild birds we sampled at two commercial poultry premises with confirmed poultry detections of H5N1 in Dubois County, IN. A total of three commercial poultry premises in Dubois County were confirmed positive for H5N1 during February 2022, and anecdotal reports confirm flocks of migrant European starlings and mixed blackbird species in the area. It is possible that the virus was present in wild bird species around these premises; however, factors in our sampling methods may have negatively impacted the ability to detect AI viruses. Surveillance began after the commercial facilities were quarantined and poultry were euthanized, potentially preventing the capture of wild birds that may have been utilizing poultry barns. Further, the approximate 2-week delay between H5N1 confirmation at the premises and the initiation of our wild bird surveillance might have contributed to the lack of detections. Other studies similarly have noted that such a delay in sampling could have decreased detection probability [18, 44]. Our study did not investigate noninfected farms, but sampling at noninfected farms in conjunction with infected farms could provide a more comprehensive view of disease ecology and host population dynamics in the area [45]. A low sample size of approximately 130 birds per farm may have influenced the ability to detect any AI viruses in captured species, and previous investigations have noted similar limitations in sample size on HPAI-infected farms [18]. Enhanced surveillance with a sufficient sample size of wild birds in known areas of HPAI virus detections in poultry and wild birds is essential to understand disease ecology and the role potential bridge hosts play in transmission [46, 47]. Conducting future sampling concurrent with poultry depopulation activities, minimizing the delay between the confirmation of HPAI and initiation of wild bird sampling, and investigating populations at uninfected farms all could provide a more comprehensive picture of wild bird-poultry transmission risk and directionality.

While this investigation suggests that synanthropic species minimally contribute to the spread of HPAI to poultry, there are inherent limiting factors that may have underrated perceived risk of transmission. Birds may die quickly once infected and their probability of capture is lower than that of healthy individuals, resulting in a potential underestimation of disease prevalence [18, 44]. Further, as passerine species tend to be smaller in size than raptors or waterfowl species, moribund and dead passerines may have a lower detection probability due to a smaller distribution of feathers and bones or quick removal from the landscape by scavengers or predators [46, 48]. Researchers determined approximately 70% of small bird carcasses experimentally placed on the landscape were removed within 24 hr by natural means and noted the presence of several scavenging species during that time frame [49]. Although rates of carcass removal are site specific and variable, evidence indicates the probability of detecting species is correlated with both the length of time postmortality and the size of the bird.

Full-length viral genome sequence analyses of 1,369 HPAI H5N1 detections in wild birds, commercial poultry, and backyard flocks from December 2021 to April 2022, suggest that at least 84% of U.S. HPAI virus detections in poultry premises and nonpoultry flocks are consistent with wild bird origin, while approximately 16% of detections are consistent with lateral transmission (poultry to poultry) [50]. This suggests that wild birds are major contributors to the spread of HPAI H5N1 to poultry, and environmental contamination or direct transmission from a variety of wild bird groups are potential sources. Further research is needed to understand transmission pathways from wild birds to poultry.

Conducting risk assessments and determining wild bird activity on farms can be used to increase biosecurity and protect domestic poultry populations [51]. Knowledge of the wild bird–poultry interface, species of concern, and the space where interspecific interactions occur is critical in developing biosecurity methods to decrease contact and risk of AI virus transmission [27]. Understanding the disease ecology and risk of viral transmission could aid producers in minimizing the risk to poultry by reducing attractants and contact between wild birds and poultry on farms. Although AI viruses previously have been detected experimentally in passerine species, including five out of the eight species sampled during targeted surveillance, results from both targeted sampling and M/M investigations in the U.S. between February 2022 and March 2023 show low prevalence in this order. More research is needed to determine which wild bird species may be involved in viral transmission to domestic poultry.

### 4.2. Morbidity/Mortality Investigations

The outbreak of HPAI H5N1 has been widespread in wild avifauna, with virus detections across the conterminous U.S. and Alaska in alternative host, synanthropic orders Accipitriformes, Cathartiformes, Falconiformes, Galliformes, Passeriformes, and Strigiformes. Prevalence rates of AI virus detections from 1,543 M/M samples tested at the NAHLN from 1 February 2022 to 31 March 2023 were highest in vultures (0.5333) followed by owls (0.2206), eagles and hawks (0.2000), falcons (0.1525), corvids (0.1240), gamebirds (0.0576), songbirds (0.0147), and doves (0.0090). Testing by the NVSL of over 2,100 samples across the same orders confirmed the presence of the HPAI EA H5 strain and suggests it was the predominant strain circulating and causing morbidities and mortalities in wild bird populations in the U.S. during this time.

Avian ecology and behavior likely play a major role in the transmission of AI viruses. Predatory and scavenging species show substantially increased levels of infection when compared to granivorous or insectivorous groups, suggesting that transmission may occur via the consumption of infected birds or mammals [37]. The order Cathartiformes, consisting of New World vultures, the only obligate scavengers sampled, had the highest rate of infection. Furthermore, roosting behavior, such as that displayed in vulture species, increases sociality between conspecifics and the likelihood of viral transmission, particularly for density-dependent pathogens such as AI viruses that spread fecal-orally [52, 53]. Facultative scavenging raptors, such as hawks, eagles, owls, and falcons, consume both carrion and apparently healthy prey, which may explain lower prevalence rates in these families. Uno et al. [54] found high levels of HPAI H5N1 infection in kestrels following experimental inoculation or ingestion of infected poultry meat. Furthermore, captive raptor morbidities and mortalities during the 2014–2015 outbreak were attributed to ingestion of infected meat [55]. Investigating families based on diet may help explain why *Corvidae*, with frequent scavenging behavior and a higher probability of feeding upon infected animals [56], have a prevalence of 0.1240 compared to approximately 0.0147 in nonomnivorous songbirds. Of all NVSL-confirmed EA H5 detections during our study period, 92% originated from raptors, indicating a potentially large role these species play in AI virus ecology. Our findings support previous research that suggests the ingestion of infected tissue is a key transmission pathway from scavenging species to conspecifics, heterospecifics, or domestic poultry [57].

Although there has been previous concern about high potential rates of infection in Galliformes due to their close association with humans and domestic poultry [58], our observed rates of infection are only slightly higher in Galliformes (0.0576) than songbirds (0.0147) and Columbiformes (0.0090). These groups have similar diets, ecological niches, and contact rates with conspecifics, humans, and domestic animals [58], suggesting that factors influencing AI transmission may go beyond physiology and behavior. Nonpredatory species tend to have increased sociality [59], but lower prevalence rates in these groups suggest that the risk of transmission by direct contact with conspecifics is low. While experimental research has indicated that AI viruses can be transmitted between species via shared environmental resources [60, 61], further research is needed to understand AI virus transmission in free-ranging avian populations.

Sampling birds as part of M/M investigations may have introduced bias into the dataset as it is more probable to detect disease in these groups than in apparently healthy birds. Further, more charismatic species such as raptors may have had disproportionate detections due to birds being larger, more noticeable, and more publicly valued. However, this methodology allowed for the largest possible dataset, potentially increasing the precision of estimates. Data collection was limited by the response rate of 67% from labs within the NAHLN as well as differences in each lab's Laboratory Information Management System taxonomy lists. Future widespread sampling of apparently healthy wildlife could provide key insights into the disease ecology of AI viruses and their implications for wildlife, human, and agricultural health.

Understanding AI virus transmission is critical to protect and manage wild bird populations, especially threatened and endangered species. Raptor species, particularly those with smaller population sizes and geographical ranges (e.g., California condors (*Gymnogyps californianus*)), that scavenge or prey upon other avian species have a higher risk of deleterious population impacts caused by HPAI virus infections [54]. Bertran et al. [62] noted that the introduction of HPAI viruses in raptors could negatively impact already threatened species and surveillance may be an invaluable tool to better understand the epidemiology of AI viruses in these populations. For example, an understanding of the increased risk for scavenging species has already been applied to management strategies meant to protect the highly endangered California condor, including vaccination and increased surveillance [63]. Monitoring sensitive species (e.g., conducting active surveillance or risk assessments) during an outbreak can offer valuable information to wildlife managers on population dynamics, disease risk, and virus type and distribution. Identifying susceptible species with fragile populations could aid in conservation efforts.

## 5. Conclusions

Conducting active surveillance of wild birds at HPAI-infected poultry facilities along with morbidity and mortality surveillance of synanthropic birds offers an avenue to better understand the ecology of AI viruses as well as the risks they pose to wildlife, domestic animals, and human health. With no active surveillance infections detected and the lowest prevalence observed between all groups sampled through morbidity and mortality investigations, orders Columbiformes and Passeriformes appear to hold less risk of AI virus infection when compared to wild predatory and scavenging avian orders, in which the most prevalent AI viral detections were found. Results of this study provide supporting evidence that consumption of infected tissues is a key transmission pathway of AI viruses. Knowing which orders and species facilitate transmission of and are more susceptible to AI virus infections can guide actions to protect domestic poultry and T&E species. Understanding factors that influence AI virus transmission is crucial for the development and implementation of superior management strategies that can help decrease the impact an HPAI outbreak has on wild and domestic bird populations.

## Figures and Tables

**Table 1:** Synanthropic bird species sampled as part of targeted surveillance at HPAI-affected commercial farms in Dubois County, Indiana, U.S., and screened for the presence of avian influenza viruses by a general influenza Type A rRT-PCR assay.

Family	Species	Birds sampled	Influenza Type A rRT-PCR detections	Prevalence	Confidence interval^a^ (lower–upper)
*Columbidae*	Eurasian collared-dove	1	0	0	0.0–0.9750
	Mourning dove	3	0	0	0.0–0.7076
	Rock dove	40	0	0	0.0–0.0881
	—	44	0	0	0.0–0.0804

*Icteridae*	Brown-headed cowbird	51	0	0	0.0–0.0698
	Common grackle	17	0	0	0.0–0.1951
	Red-winged blackbird	13	0	0	0.0–0.2471
	—	81	0	0	0.0–0.0445

*Passeridae*	House sparrow	89	0	0	0.0–0.0406
	—	89	0	0	0.0–0.0406

*Sturnidae*	European starling	52	0	0	0.0–0.0685
	—	52	0	0	0.0–0.0685

Total	—	266	0	0	0.0–0.0138

^a^All confidence intervals were calculated using a 0.95 confidence level.

**Table 2:** Number of confirmed HPAI EA H5 and EA H5N1 detections by avian order as determined at the NVSL by rRT-PCR assay targeting Eurasian lineage Gs/GD H5 clade 2.3.4.4b from 1 February 2022 to 31 March 2023.

Order	H5 2.3.4.4b rRT-PCR confirmed detections
Accipitriformes	840
Cathartiformes	671
Falconiformes	61
Galliformes	30
Passeriformes	149
Strigiformes	370

Total	2,121

## Data Availability

Data for highly pathogenic avian influenza detections in wild birds confirmed at the NVSL from 2022 to 2024 are available at the following link: https://www.aphis.usda.gov/livestock-poultry-disease/avian/avian-influenza/hpai-detections/wild-birds (USDA APHIS | 2022−2024 Detections of Highly Pathogenic Avian Influenza in Wild Birds). The majority of data supporting this research are restricted and not available publicly. Wild bird targeted surveillance influenza data collected between August 2007 and March 2024 are available from the Wildlife Services National Wildlife Disease Program (NWDP) of the USDA by contacting the NWDP at nwdpdata@usda.gov.

## Supplementary Materials

Supplementary Materials Compiled dataset of all families, species, number of birds sampled, positive detections, and confidence intervals as determined by general influenza Type A rRT-PCR assay across all samples tested at diagnostic laboratories in the NAHLN from 1 February 2022 to 31 March 2023 as part of morbidity/mortality events.
