# Supplementary material for: Prevalence of Avian Influenza Virus in Atypical Wild Birds Host Groups during an Outbreak of Highly Pathogenic Strain EA/AM H5N1
Source: Transbound Emerg Dis. 2024 Jul 29;2024:4009552. doi: 10.1155/2024/4009552 (PMC12016917; doi:10.1155/2024/4009552)
Supplement: Supplementary Materials — Compiled dataset of all families, species, number of birds sampled, positive detections, and confidence intervals as determined by general influenza Type A rRT-PCR assay across all samples tested at diagnostic laboratories in the NAHLN from 1 February 2022 to 31 March 2023 as part of morbidity/mortality events. [file 4009552.f1.docx]

Table S1. All species sampled as part of morbidity/mortality events and screened for the presence of avian influenza viruses by a general influenza Type A rRT-PCR assay at diagnostic laboratories in the NAHLN from 1 February 2022 to 31 March 2023.

| Order | Family | Species | Scientific Name | Birds Sampled | Influenza Type A rRT-PCR Detections | Prevalence | Confidence Interval^1^  (Lower – Upper) |
| --- | --- | --- | --- | --- | --- | --- | --- |
| **Columbiformes** | **Total** |  |  | **443** | **4** | **0.0090** | **0.0025 – 0.0230** |
|  |  | Common ground dove | *Columbina passerina* | 4 | 0 | 0 | 0.0 – 0.6024 |
|  |  | Eurasian collared dove | *Streptopelia decaocto* | 11 | 0 | 0 | 0.0 – 0.2849 |
|  |  | Mourning dove | *Zenaida macroura* | 92 | 2 | 0.0217 | 0.0026 – 0.0763 |
|  |  | Rock dove | *Columba livia* | 244 | 2 | 0.0082 | 0.0010 – 0.0293 |
|  |  | White-winged dove | *Zenaida asiatica* | 3 | 0 | 0 | 0.0 – 0.7076 |
|  |  | Dove (unidentified) |  | 54 | 0 | 0 | 0.0 – 0.0660 |
|  |  | Pigeon (unidentified) |  | 35 | 0 | 0 | 0.0 – 0.1000 |
| Columbiformes | *Columbidae* |  |  | 443 | 4 | 0.009 | 0.0025 – 0.0230 |
| **Passeriformes** | **Total** |  |  | **1417** | **79** | **0.0558** | **0.0440 – 0.0690** |
|  |  | Cedar waxwing | *Bombycilla cedrorum* | 20 | 0 | 0 | 0.0 – 0.1684 |
|  |  | Bohemian waxwing | *Bombycilla garrulus* | 1 | 0 | 0 | 0.0 – 0.9750 |
| Passeriformes | *Bombycillidae* |  |  | 21 | 0 | 0 | 0.0 – 0.1611 |
|  |  | Northern cardinal | *Cardinalis cardinalis* | 9 | 0 | 0 | 0.0 – 0.3363 |
|  |  | Indigo bunting | *Passerina cyanea* | 3 | 0 | 0 | 0.0 – 0.7076 |
|  |  | Rose-breasted grosbeak | *Pheucticus ludovicianus* | 2 | 0 | 0 | 0.0 – 0.8419 |
|  |  | Scarlet tanager | *Piranga olivacea* | 2 | 0 | 0 | 0.0 – 0.8419 |
| Passeriformes | *Cardinalidae* |  |  | 16 | 0 | 0 | 0.0 – 0.2059 |
|  |  | Common redpoll | *Acanthis flammea* | 2 | 0 | 0 | 0.0 – 0.8419 |
|  |  | House finch | *Haemorhous mexicanus* | 10 | 0 | 0 | 0.0 – 0.3085 |
|  |  | Evening grosbeak | *Hesperiphona vespertina* | 1 | 0 | 0 | 0.0 – 0.9750 |
|  |  | Pine grosbeak | *Pinicola enucleator* | 4 | 1 | 0.25 | 0.0063 – 0.8059 |
|  |  | American goldfinch | *Spinus tristis* | 19 | 1 | 0.0526 | 0.0013 – 0.2603 |
|  |  | Finch (unidentified) |  | 11 | 0 | 0 | 0.0 – 0.2849 |
| Passeriformes | *Fringillidae* |  |  | 47 | 2 | 0.0426 | 0.0052 – 0.1454 |
|  |  | Barn swallow | *Hirundo rustica* | 12 | 0 | 0 | 0.0 – 0.2646 |
|  |  | Tree swallow | *Tachycineta bicolor* | 19 | 2 | 0.1053 | 0.0130 – 0.3314 |
|  |  | Violet-green swallow | *Tachycineta thalassina* | 4 | 3 | 0.75 | 0.1941 – 0.9937 |
| Passeriformes | *Hirundinidae* |  |  | 35 | 5 | 0.1429 | 0.0481 – 0.3026 |
|  |  | Boat-tailed grackle | *Quiscalus major* | 1 | 1 | 1 | 0.0 – 0.9750 |
|  |  | Bobolink | *Dolichonyx oryzivorus* | 1 | 0 | 0 | 0.0 – 0.9750 |
|  |  | Brown-headed cowbird | *Molothrus ater* | 7 | 0 | 0 | 0.0 – 0.4096 |
|  |  | Common grackle | *Quiscalus quiscula* | 95 | 1 | 0.0105 | 0.0003 – 0.0573 |
|  |  | Red-winged blackbird | *Agelaius phoeniceus* | 9 | 1 | 0.1111 | 0.0028 – 0.4825 |
|  |  | Blackbird (unidentified) |  | 4 | 0 | 0 | 0.0 – 0.6024 |
| Passeriformes | *Icteridae* |  |  | 120 | 3 | 0.025 | 0.0052 – 0.0713 |
|  |  | Brown thrasher | *Toxostoma rufum* | 1 | 0 | 0 | 0.0 – 0.9750 |
|  |  | Curve-billed thrasher | *Toxostoma curvirostre* | 1 | 0 | 0 | 0.0 – 0.9750 |
|  |  | Gray catbird | *Dumetella carolinensis* | 9 | 0 | 0 | 0.0 – 0.3363 |
|  |  | Northern mockingbird | *Mimus polyglottos* | 1 | 0 | 0 | 0.0 – 0.9750 |
| Passeriformes | *Mimidae* |  |  | 12 | 0 | 0 | 0.0 – 0.2646 |
| Passeriformes | *Oriolodae* | Oriole (unidentified) |  | 7 | 0 | 0 | 0.0 – 0.4096 |
|  |  | Black-capped chickadee | *Poecile atricapillus* | 26 | 0 | 0 | 0.0 – 0.1323 |
|  |  | Tufted titmouse | *Baeolophus bicolor* | 2 | 0 | 0 | 0.0 – 0.8419 |
| Passeriformes | *Paridae* |  |  | 28 | 0 | 0 | 0.0 – 0.1234 |
|  |  | Common yellowthroat | *Geothlypis trichas* | 1 | 0 | 0 | 0.0 – 0.9750 |
|  |  | Magnolia warbler | *Setophaga magnolia* | 15 | 0 | 0 | 0.0 – 0.2180 |
|  |  | Orange-crowned warbler | *Leiothlypis celata* | 1 | 0 | 0 | 0.0 – 0.9750 |
|  |  | Yellow warbler | *Setophaga petechia* | 2 | 0 | 0 | 0.0 – 0.8419 |
|  |  | Warbler (unidentified) |  | 13 | 0 | 0 | 0.0 – 0.2471 |
| Passeriformes | *Parulidae* |  |  | 32 | 0 | 0 | 0.0 – 0.1089 |
|  |  | Dark-eyed junco |  | 19 | 1 | 0.0526 | 0.0013 – 0.2603 |
|  |  | Song sparrow | *Melospiza melodia* | 1 | 0 | 0 | 0.0 – 0.9750 |
|  |  | Fox sparrow | *Passerella iliaca* | 2 | 0 | 0 | 0.0 – 0.8419 |
|  |  | Spotted towhee | *Pipilo maculatus* | 1 | 0 | 0 | 0.0 – 0.9750 |
|  |  | White-throated sparrow | *Zonotrichia albicollis* | 7 | 0 | 0 | 0.0 – 0.4096 |
| Passeriformes | *Passerellidae* |  |  | 30 | 1 | 0.0333 | 0.0008 – 0.1722 |
| Passeriformes | *Passeridae* | House sparrow | *Passer domesticus* | 165 | 1 | 0.0061 | 0.0002 – 0.0333 |
|  |  | Nuthatch (unidentified) |  | 3 | 0 | 0 | 0.0 – 0.7076 |
|  |  | White-breasted nuthatch | *Sitta carolinensis* | 1 | 0 | 0 | 0.0 – 0.9750 |
| Passeriformes | *Sittidae* |  |  | 4 | 0 | 0 | 0.0 – 0.6024 |
| Passeriformes | *Sturnidae* | European starling | *Sturnus vulgaris* | 72 | 0 | 0 | 0.0 – 0.0499 |
| Passeriformes | *Thraupidae* | Tanager (unidentified) |  | 3 | 0 | 0 | 0.0 – 0.7076 |
|  |  | Carolina wren | *Thryothorus ludovicianus* | 11 | 0 | 0 | 0.0 – 0.2849 |
|  |  | Pacific wren | *Troglodytes pacificus* | 1 | 0 | 0 | 0.0 – 0.9750 |
|  |  | Wren (unidentified) |  | 2 | 0 | 0 | 0.0 – 0.8419 |
| Passeriformes | *Troglodytidae* |  |  | 14 | 0 | 0 | 0.0 – 0.2316 |
|  |  | American robin | *Turdus migratorius* | 170 | 1 | 0.0059 | 0.0001 – 0.0323 |
|  |  | Eastern bluebird | *Sialia sialis* | 31 | 0 | 0 | 0.0 – 0.1122 |
|  |  | Hermit thrush | *Catharus guttatus* | 2 | 0 | 0 | 0.0 – 0.8419 |
|  |  | Swainson’s thrush | *Catharus ustulatus* | 2 | 0 | 0 | 0.0 – 0.8419 |
|  |  | Thrush (unidentified) |  | 9 | 0 | 0 | 0.0 – 0.3363 |
|  |  | Townsend's solitaire | *Myadestes townsendi* | 1 | 0 | 0 | 0.0 – 0.9750 |
|  |  | Varied thrush | *Ixoreus naevius* | 20 | 0 | 0 | 0.0 – 0.1684 |
|  |  | Veery | *Catharus fuscescens* | 1 | 0 | 0 | 0.0 – 0.9750 |
|  |  | Wood thrush | *Hylocichla mustelina* | 14 | 0 | 0 | 0.0 – 0.2316 |
| Passeriformes | *Turdidae* |  |  | 250 | 1 | 0.004 | 0.0001 – 0.0221 |
| Passeriformes | *Tyrannidae* | Eastern phoebe | *Sayornis phoebe* | 12 | 0 | 0 | 0.0 – 0.2646 |
| Passeriformes | *Vireonidae* | Red-eyed vireo | *Vireo olivaceus* | 2 | 0 | 0 | 0.0 – 0.8419 |
|  |  | American crow | *Corvus brachyrhynchos* | 301 | 30 | 0.0997 | 0.0683 – 0.1392 |
|  |  | Black-billed magpie | *Pica hudsonia* | 35 | 6 | 0.1714 | 0.0656 – 0.3365 |
|  |  | Blue jay | *Cyanocitta cristata* | 39 | 0 | 0 | 0.0 – 0.0903 |
|  |  | Common raven | *Corvus corax* | 106 | 25 | 0.2358 | 0.1588 – 0.3282 |
|  |  | Crow (unidentified) |  | 8 | 0 | 0 | 0.0 – 0.3694 |
|  |  | Fish crow | *Corvus ossifragus* | 24 | 5 | 0.2083 | 0.0713 – 0.4215 |
|  |  | Gray jay | *Perisoreus canadensis* | 1 | 0 | 0 | 0.0 – 0.9750 |
|  |  | Green jay | *Cyanocorax luxuosus* | 3 | 0 | 0 | 0.0 – 0.7076 |
|  |  | Magpie (unidentified) |  | 7 | 0 | 0 | 0.0 – 0.4096 |
|  |  | Scrub jay (unidentified) |  | 3 | 0 | 0 | 0.0 – 0.7076 |
|  |  | Steller's jay | *Cyanocitta stelleri* | 5 | 0 | 0 | 0.0 – 0.5218 |
| Passeriformes | *Corvidae* |  |  | 532 | 66 | 0.124 | 0.0973 – 0.1551 |
| **Piciformes** | **Total** |  |  | **15** | **0** | **0** | **0.0 – 0.2180** |
|  |  | Northern flicker | *Colaptes auratus* | 11 | 0 | 0 | 0.0 – 0.2849 |
|  |  | Red-breasted sapsucker | *Sphyrapicus ruber* | 1 | 1 | 0 | 0.0 – 0.9750 |
|  |  | Yellow-bellied sapsucker | *Sphyrapicus varius* | 1 | 0 | 0 | 0.0 – 0.9750 |
|  |  | Woodpecker (unidentified) |  | 2 | 0 | 0 | 0.0 – 0.8419 |
| Piciformes | *Picidae* |  |  | 15 | 0 | 0 | 0.0 – 0.2180 |
| **Accipitriformes** | **Total** |  |  | **2850** | **570** | **0.2000** | **0.1855 – 0.2152** |
|  |  | Bald eagle | *Haliaeetus leucocephalus* | 1150 | 294 | 0.2557 | 0.2307 – 0.2819 |
|  |  | Broad-winged hawk | *Buteo platypterus* | 120 | 6 | 0.05 | 0.0186 – 0.1057 |
|  |  | Coopers hawk | *Accipiter cooperii* | 279 | 22 | 0.0789 | 0.0501 – 0.1170 |
|  |  | Ferruginous hawk | *Buteo regalis* | 1 | 0 | 0 | 0.0 – 0.9750 |
|  |  | Golden eagle | *Aquila chrysaetos* | 69 | 3 | 0.0435 | 0.0091 – 0.1218 |
|  |  | Harris's hawk | *Parabuteo unicinctus* | 27 | 0 | 0 | 0.0 – 0.1277 |
|  |  | Mississippi kite | *Ictinia mississippiensis* | 11 | 0 | 0 | 0.0 – 0.2849 |
|  |  | Northern goshawk | *Accipiter gentilis* | 2 | 0 | 0 | 0.0 – 0.8419 |
|  |  | Norther harrier | *Circus hudsonius* | 2 | 0 | 0 | 0.0 – 0.8419 |
|  |  | Red-shouldered hawk | *Buteo lineatus* | 179 | 20 | 0.1117 | 0.0696 – 0.1673 |
|  |  | Red-tailed hawk | *Buteo jamaicensis* | 747 | 193 | 0.2584 | 0.2273 – 0.2913 |
|  |  | Rough-legged hawk | *Buteo lagopus* | 16 | 8 | 0.5 | 0.2465 – 0.7535 |
|  |  | Sharp-shinned hawk | *Accipiter striatus* | 55 | 4 | 0.0727 | 0.0202 – 0.1759 |
|  |  | Swainson's hawk | *Buteo swainsoni* | 19 | 4 | 0.2105 | 0.0605 – 0.4557 |
|  |  | Hawk (unidentified) |  | 78 | 10 | 0.1282 | 0.0632 – 0.2232 |
|  |  | Eagle (unidentified) |  | 13 | 2 | 0.1538 | 0.0192 – 0.4545 |
| Accipitriformes | *Accipitridae* |  |  | 2768 | 566 | 0.2044 | 0.1896 – 0.2200 |
| Accipitriformes | *Pandionidae* | Osprey | *Pandion haliaetus* | 82 | 4 | 0.0488 | 0.0134 – 0.1202 |
| **Cathartiformes** | **Total** |  |  | **795** | **424** | **0.5333** | **0.4980 – 0.5685** |
|  |  | Black vulture | *Coragyps atratus* | 495 | 336 | 0.6788 | 0.6357 – 0.7198 |
|  |  | California condor | *Gymnogyps californianus* | 80 | 3 | 0.0375 | 0.0078 – 0.1057 |
|  |  | Turkey vulture | *Cathartes aura* | 186 | 73 | 0.3925 | 0.3218 – 0.4666 |
|  |  | *Cathartidae* (unidentified) |  | 34 | 12 | 0.3529 | 0.1975 – 0.5351 |
| Cathartiformes | *Cathartidae* |  |  | 795 | 424 | 0.5333 | 0.4980 – 0.5685 |
| **Falconiformes** | **Total** |  |  | **387** | **59** | **0.1525** | **0.1181 – 0.1922** |
|  |  | American kestrel | *Falco sparverius* | 101 | 3 | 0.0297 | 0.0062 – 0.0844 |
|  |  | Aplomado falcon | *Falco femoralis* | 2 | 0 | 0 | 0.0 – 0.8419 |
|  |  | Gyrfalcon | *Falco rusticolus* | 80 | 0 | 0 | 0.0 – 0.0451 |
|  |  | Merlin | *Falco columbarius* | 46 | 4 | 0.087 | 0.0242 – 0.2079 |
|  |  | Peregrine falcon | *Falco peregrinus* | 148 | 46 | 0.3108 | 0.2374 – 0.3920 |
|  |  | Prairie falcon | *Falco mexicanus* | 1 | 1 | 1 | 0.0 – 0.9750 |
|  |  | *Falconidae* (unidentified) |  | 9 | 5 | 0.5556 | 0.2120 – 0.8630 |
| Falconiformes | *Falconidae* |  |  | 387 | 59 | 0.1525 | 0.1181 – 0.1922 |
| **Strigiformes** | **Total** |  |  | **1274** | **281** | **0.2206** | **0.1981 – 0.2444** |
|  |  | Barred owl | *Strix varia* | 320 | 23 | 0.0719 | 0.0461 – 0.1059 |
|  |  | Boreal owl | *Aegolius funereus* | 1 | 0 | 0 | 0.0 – 0.9750 |
|  |  | Burrowing owl | *Athene cunicularia* | 7 | 0 | 0 | 0.0 – 0.4096 |
|  |  | Eastern screech-owl | *Magascops asio* | 96 | 3 | 0.0313 | 0.0065 – 0.0886 |
|  |  | Flammulated owl | *Psiloscops flammeolus* | 5 | 0 | 0 | 0.0 – 0.5218 |
|  |  | Great grey owl | *Strix nebulosa* | 4 | 0 | 0 | 0.0 – 0.6024 |
|  |  | Great horned owl | *Bubo virginianus* | 610 | 234 | 0.3836 | 0.3448 – 0.4235 |
|  |  | Long-eared owl | *Asio otus* | 11 | 1 | 0.0909 | 0.0023 – 0.4128 |
|  |  | Northern hawk-owl | *Surnia ulula* | 1 | 0 | 0 | 0.0 – 0.9750 |
|  |  | Northern saw-whet owl | *Aegolius acadicus* | 5 | 0 | 0 | 0.0 – 0.5218 |
|  |  | Short-eared owl | *Asio flammeus* | 3 | 1 | 0.3333 | 0.0084 – 0.9057 |
|  |  | Snowy owl | *Bubo scandiacus* | 28 | 9 | 0.3214 | 0.1588 – 0.5235 |
|  |  | Spotted owl | *Strix occidentalis* | 1 | 0 | 0 | 0.0 – 0.9750 |
|  |  | Western screech owl | *Megascops kennicottii* | 5 | 0 | 0 | 0.0 – 0.5218 |
|  |  | *Strigidae* (unidentified) |  | 116 | 10 | 0.0862 | 0.0421 – 0.1528 |
| Strigiformes | *Strigidae* |  |  | 1213 | 281 | 0.2317 | 0.2082 – 0.2564 |
| Strigiformes | *Tytonidae* | Barn owl |  | 61 | 0 | 0 | 0.0 – 0.0587 |
| **Galliformes** | **Total** |  |  | **2187** | **126** | **0.0576** | **0.0482 - 0.0682** |
|  |  | Greater sage grouse | *Centrocercus urophasianus* | 5 | 1 | 0.2 | 0.0051 – 0.7164 |
|  |  | Grouse (unidentified) |  | 10 | 0 | 0 | 0.0 – 0.3085 |
|  |  | Pheasant (unidentified) |  | 674 | 61 | 0.0905 | 0.0699 – 0.1147 |
|  |  | Ring-necked pheasant | *Phasianus colchicus* | 233 | 31 | 0.133 | 0.0922 – 0.1835 |
|  |  | Ruffed grouse | *Bonasa umbellus* | 15 | 1 | 0.0667 | 0.0017 – 0.3195 |
|  |  | Sharp-tailed grouse | *Tympanuchus phasianellus* | 40 | 0 | 0 | 0.0 – 0.0881 |
|  |  | Spruce grouse | *Canachites canadensis* | 1 | 0 | 0 | 0.0 – 0.9750 |
|  |  | Wild turkey | *Meleagris gallopavo* | 451 | 29 | 0.0643 | 0.0435 – 0.0910 |
|  |  | Willow ptarmigan | *Lagopus lagopus* | 1 | 0 | 0 | 0.0 – 0.9750 |
| Galliformes | *Phasianidae* |  |  | 1430 | 123 | 0.086 | 0.0720 – 0.1018 |
|  |  | Northern bobwhite quail | *Colinus virginianus* | 56 | 0 | 0 | 0.0 – 0.0638 |
|  |  | Quail (unidentified) |  | 701 | 3 | 0.0040 | 0.0009 – 0.0125 |
| Galliformes | *Odontophoridae* |  |  | 757 | 3 | 0.0039 | 0.0008 – 0.0115 |

^1^All confidence intervals were calculated using a .95 confidence level.
